# From “combined prevention” to “comprehensive prevention”: building the response to the syndemic with adolescents and youth in São Paulo, Brazil (2020-2023)

**DOI:** 10.1590/0102-311XEN084323

**Published:** 2025-05-19

**Authors:** Vera Paiva, José Ricardo de C. M. Ayres, Ivan França, Marcos Roberto Vieira Garcia, Cristiane Gonçalves da Silva, Júlio Assis Simões, Luis Guilherme Galeao-Silva, Jan Billand, Grupo de Pesquisa sobre Saúde dos Jovens e Direitos Humanos

**Affiliations:** 1 Instituto de Psicologia, Universidade de São Paulo, São Paulo, Brasil.; 2 Faculdade de Medicina, Universidade de São Paulo, São Paulo, Brasil.; 3 Faculdade de Saúde Pública, Universidade de São Paulo, São Paulo, Brasil.; 4 Universidade Federal de São Carlos, Sorocaba, Brasil.; 5 Universidade Federal de São Paulo, Santos, Brasil.; 6 Faculdade de Filosofia, Letras e Ciências Humanas, Universidade de São Paulo, São Paulo, Brasil.

**Keywords:** Adolescent, Disease Prevention, Integrality in Health, COVID-19, HIV, Adolescente, Prevención de Enfermedades, Integralidad en Salud, COVID-19, VIH

## Abstract

This essay discusses the need for and the possibilities of transitioning from the conceptual framework of combined prevention to the conception of “comprehensive prevention,” based on intervention research aimed at supporting and protecting the sexual and reproductive health of adolescents and youth in three cities in the state of São Paulo, Brazil (São Paulo, Santos and Sorocaba). Particularly effective in preparing for syndemic cycles within the context of environmental crises, the concept of comprehensive prevention contributed to understanding processes of vulnerability to COVID-19, mpox, sexually transmitted infections/AIDS, violence, unwanted pregnancy and psychosocial distress. It provided guidance for addressing the syndemic nature of pandemics intensified by social crises, and encouraged young people to develop creative responses to political polarization fueled by the far right, which aggressively inhibits approaches based on human rights, especially in the field of sexuality, enhancing the infodemic that downplays the severity of ongoing pandemics. We underline the productivity of the concept of comprehensive care in developing initiatives related to human rights and prevention which dynamically anticipate and address events that synergistically overlap in marginalized territories. By adopting “scenes” as units of interpretation and focal points for responding to contexts of vulnerability, we favor the co-construction of transversal skills in the personal, collective and territorial responses necessary for the prevention of adversities that threaten adolescents and youth both physically and mentally.

## Abstract (es)

Este ensayo discute la necesidad y las posibilidades de pasar de la orientación conceptual de la prevención combinada a la concepción de “prevención integral”, a partir de experiencias de investigación-intervención en la promoción y protección de la salud sexual y reproductiva entre adolescentes y jóvenes en tres ciudades del estado de São Paulo, Brasil (São Paulo, Santos y Sorocaba). Eficaz en la preparación para ciclos sindémicos en el contexto de crisis ambiental, la prevención integral ha favorecido la inteligibilidad de los procesos de vulnerabilidad al COVID-19 y a la mpox, a las enfermedades de transmisión sexual/sida, a la violencia, al embarazo no deseado y al sufrimiento psicosocial. También ha permitido orientar las respuestas a la sindemia pandémica en el contexto de la crisis social que la había intensificado y ha estimulado a los jóvenes a dar respuestas creativas en el contexto de la polarización política promovida por la extrema derecha, que impide agresivamente los derechos humanos, especialmente en el campo de la sexualidad, exacerbando la infodemia que descalifica la gravedad de las pandemias en curso. La productividad del concepto de integralidad de la atención se destaca para establecer acciones en el contexto de los derechos humanos y la prevención que anticipen y aborden dinámicamente eventos que se superponen de forma sinérgica en territorios periféricos. La valoración de las “escenas” como unidad interpretativa y foco de respuestas a contextos de vulnerabilidad permite favorecer la coconstrucción de habilidades transversales en las respuestas personales, colectivas y territoriales necesarias para prevenir enfermedades que afectan concomitantemente la salud física y mental de adolescentes y jóvenes.

## Introduction

Since the 1990s, through different outreach and research projects in the fields of sexuality, youth rights promotion and AIDS prevention ^1^ ^,^ ^2^ ^,^ ^3^ ^,^ ^4^ ^,^ ^5^ ^,^ ^6^ ^,^ ^7^, we have celebrated the enjoyment - and not just the risks - of sexual and gender diversity, and different religious cultures, safeguarded by State secularism as provided by the *Brazilian Federal Constitution* promulgated at the time. In the state of São Paulo, besides concerns about sexually transmited infections (STIs)/AIDS, we have addressed the persistent interest of both adults and youth in pregnancy prevention.

At the turn of the 21st century, in the territories where we continued to promote sexual and reproductive health, we worked with adolescents and youth growing up in families affiliated with evangelical Christian churches. Many of them were newly converted and committed to preaching their values and sexual morality, often engaging in the “demonization” of other religious traditions, an uncommon practice since the return to democracy in Brazil. Drawing on the vulnerability and human rights framework ^8^ ^,^ ^9^, we examined the role of religion in responding to AIDS and, since 2010, have tracked the retrograde public policies advocated by politicians who exploited fundamentalist religious rhetoric, both Evangelical and Catholic, to defend “*the inherent nature of the body*” and decry what they labeled as “*gender ideology*” ^10^ ^,^ ^11^ ^,^ ^12^ ^,^ ^13^.

Using intervention research, we demonstrated how the territories where adolescents and youth live, study and socialize define particularities that are indispensable for understanding and mitigating vulnerability to illness ^6^ ^,^ ^7^ ^,^ ^14^ ^,^ ^15^. Territories have history and culture, and in peripheral areas they define communities based on shared worldviews and processes of political resistance against exclusion, stigmatization, gender- and sexuality-based violence, and psychological distress. Territorial connections guide the establishment of public service centers within Brazilian Unified National Health System (SUS, acronym in Portuguese) and Brazilian Unified Social Assistance System (SUAS, acronym in Portuguese), according to principles of regionalization and decentralization. This territorial framework also influences the location of Christian churches, particularly neo-Pentecostal ones.

Social and political action in such territories shapes the possibilities for maintaining community health and the opportunities for mitigating vulnerabilities to illness, operating within a delicate balance between domination (exploitation for profit) and appropriation (survival and social and cultural production) ^16^.

The election of Jair Bolsonaro as Brazilian president in 2018 and the subsequent rise of overtly far-right conservative state governments accelerated the setbacks. Between 2010 and 2021, without access to comprehensive human rights-based sexual education and prevention, adolescents and youth vulnerability increased: the AIDS detection rate (per 100,000 inhabitants) in the 15-24 age group rose from 11.5 to 13.3, while condom use declined with no significant increase in the number of adolescents and youth using HIV pre-exposure prophylaxis (PrEP) provided by the SUS ^17^.

In 2020, as the world came to a halt due to the severity of the COVID-19 pandemic, we trained high school students as youth agents of research and prevention of STIs/AIDS and pregnancy. In addition, by using intersectional methodologies, we explored the impact of stigma and rights violation as part of an intervention research project (*Youth Vulnerabilities to STIs/HIV and Intimate Partner Violence: Evaluation of Psychosocial Human Rights-Based Intervention*) ^18^.

We developed this project in a challenging context, responding to the dynamics of two simultaneous crises.

(1) Progress of the syndemic of infectious diseases - COVID-19, STIs (syphilis and AIDS), mpox, dengue fever - and psychosocial distress caused by socio-political crises that aggravate these issues. As argued by Singer et al. ^19^ a syndemic approach examines the pathways that create synergy between diseases, amplifying harm, and how inequality and injustice contribute to their interaction and to social vulnerability. In 2020, Horton ^20^ highlighted the syndemic nature of COVID-19, emphasizing the need for a multi-sectoral response through initiatives in health and education, as well as policies on housing, employment, food security and the environment, especially when integrated at a “local” level, which we understand as “territorial”;

(2) Political polarization, the aggressive political strategy of the far-right to heighten tensions, polarizing everything and everyone to spread rumors and disinformation on social media. Such political divisiveness generates conservative “anti-agendas” in the fields of AIDS ^21^ and reproductive health, which not only inhibit approaches grounded in human rights but also intensify the infodemic. Such manipulation of information is intentionally aimed at obstructing access to and validation of reliable sources and guidance on prevention.

We strove to implement and evaluate a model of combined HIV/AIDS prevention in eight high schools, integrated with health centers within the same territories in the cities of São Paulo, Santos and Sorocaba. Therefore, our goal was “*to reduce new HIV infections by combining biomedical, behavioral and structural strategies based on human rights and evidence, considering local contexts*” ^22^.

Among the various lessons learned from this experience, spanning from 2019 to 2023, we will highlight in this essay the conceptual shifts required to overcome the “*fractional universalization*” ^23^ of the most commonly adopted prevention proposals, even within the framework of combined prevention. The mere offer of a “*basket*” of preventive options to be “*consumed*” ^5^ at each person’s convenience, along the lines of a given neoliberal ideology ^24^, strikes us as insufficient - if not an outright obstacle - for the development of effective health promotion practices and adequate preparation to address the new syndemic cycles we will continue to face in the coming years.

The aim of this paper is to discuss the need for, and point out the possibilities of, transitioning from the conceptual framework of combined prevention to the concept of comprehensive prevention in the development of initiatives to promote and protect sexual and reproductive health in syndemic contexts, drawing mainly on the experiences of territory-based intervention research conducted by the authors.

## Adapting research and intervention methodologies “in times of the zombie apocalypse”

Impacted by COVID-19, the intervention research project that inspires this essay required refining the process of securing ongoing consent from the school communities in the eight peripheral neighborhoods and favelas selected for the study. Each change in context and design also required approval from the Research Ethics Committee of the Institute of Psychology, University of São Paulo (USP; protocols: n. 2,979,702 on October 24, 2018; n. 4,079,347 on June 9, 2020; and n. 5.334.405 on April 6, 2022). After institutional consent had been obtained in the three cities, the first step was to invite students from both technical and regular high schools to take part in the project.

No significant differences were observed in the profiles of participating students over the study period. In 2019, 719 12th-grade students took a questionnaire designed to identify their vulnerabilities (personal, programmatic and social) to STIs, intimate partner and peer violence, and psychosocial distress. They accessed the questions on tablets and computers provided at school. In 2020-2021, in collaboration with youth agents and teachers, with interactions continued online, we responded to the COVID-19 emergency by designing and implementing research and prevention activities in the territories. These activities, accompanied by ethnographic records, informed the reconstruction of the questionnaire and the approach to its administration. In 2022, 1,242 12th-grade students, who were graduating after attending high school during the most severe years of COVID-19, completed the updated questionnaire in person. This version included questions about their experiences during the pandemic. The majority self-identified as white or Asian (52%), while 48% self-identified as black, brown or Indigenous. A high percentage reported having “no religion” (44%), and Catholics accounted for 20%, Pentecostal Evangelicals for 17% and practitioners of Umbanda/Candomblé for 5%, while 5% indicated other affiliations or chose not to respond. Approximately 44% self-identified as male, 52% as female and 5% chose the option “I self-identify differently”, neither “male” nor “female”. Among the respondents, 20% of boys and 45% of girls self-identified as gay/lesbian/pansexual/another orientation/questioning.

The research team - interprofessional, interdisciplinary and intergenerational - included 240 high school students trained as youth agents between 2020 and 2023, and continued to engage with the evolving context, aiming to inform public policies grounded in human rights. It acknowledged that adolescents and youth have different social classes, races/ethnicities, genders, sexualities and religious beliefs, and that such differences lead to inequalities.

Since the preliminary study, whose results were discussed in online meetings and conversation circles with the youth agents and teachers from the eight school territories involved in the project, we had already identified the setbacks in promoting and protecting sexual health resulting from the rise of conservative governments and the neoliberal abandonment of social policies ^24^. Like other Brazilian studies on combined prevention among youth ^25^, we observed how social vulnerability to illness reflected the daily lives of youth in these territories, marked by inequality.

Political polarization, which increased during electoral periods, mobilized parents opposed to the project and conservative teachers, often silencing educators who feared aggressive backlash against the school. It surfaced in provocative comments during debates to present the questionnaire results and in the timid resistance of teachers and school administrators to the surprising denialism of some science teachers. Nevertheless, the creativity of school administrators and students in striving to continue the intervention research enabled us to face the impacts and persistent legacy of the pandemic, which continues to disproportionately affect a generation of adolescents and youth ^26^ ^,^ ^27^ ^,^ ^28^.

The aggressive acts of the far-right, which facilitated the spread of the virus, are well-documented and analyzed ^29^ ^,^ ^30^. Especially during the period discussed in this paper, the lack of leadership and coordination among federal, state and municipal governments left a historical burden for adolescents and youth, which we term the long-term impact of COVID-19. This includes psychosocial effects that extend beyond the current biomedical definitions of long COVID.

The differences in responses to the AIDS and COVID-19 epidemics are striking. The adverse and complex conditions during the COVID-19 emergency - a disease transmitted through the air rather than solely through the exchange of bodily fluids, like STIs - and the significant impact on morbidity and mortality required a much larger scale of responses. At the same time, during the construction of Brazil’s response to AIDS, the country was emerging from a dictatorship and witnessing the defeat of the violent far-right forces that supported it, in a context of increasing democratization that benefitted from the enthusiastic collaboration of generations across various sectors of society, dedicated to building a welfare state and policies based on human rights.

The legacy of the AIDS response included a consensus on the social and structural determinants of pandemics and the importance of community organization in producing effective responses to control them. Parker & Camargo Jr. ^31^ highlighted the synergistic effect of structural determinants and social exclusion in HIV infection and AIDS when they discussed the “*synergy of plagues*” and “*AIDS as a syndemic*”, as defined by Singer.

Regarding the psychosocial response involved in prevention efforts and individual experiences, we had also learned from the AIDS epidemic that: (a) fear can amplify the denial of risk and the stigmatization of at-risk groups; (b) social solidarity is essential for tackling local epidemics; and (c) changing behaviors and social interaction practices is always a complex and multifaceted process that requires time for people to internalize their experience of self-care and care for others, evaluating and adjusting it to the dynamics of each situation of vulnerability.

In the response to COVID-19, in turn, the role of youth in vulnerability assessments shifted from being part of the “*highest at-risk group*” during the HIV/AIDS pandemic to a group perceived as less susceptible (compared to older populations), an argument repeatedly used by governments and amplified by the media. At the same time, the increased digital connectivity among youth in peripheral areas and universities allowed them to continue their education without in-person classes and supported our collaboration to respond to the crisis. High school students and small research teams assigned to each school territory kept in touch by phone or online, often interacting with educational coordinators, school principals and teaching staff involved in the project to discuss and consolidate analyses of what we co-observed in each territory.

For the first 18 months, such conversation and listening depended on this interaction conducted from our homes, where we were all isolated, mediated by screens and internet access - which was of poor quality for youth living in peripheral areas ^32^. Paulo Freire’s concept of decoding words into praxis was adapted to decoding daily situations of exposure to various hardships affecting the lives of adolescents and youth, seeking local meanings and senses that enabled the coproduction of prevention practices ^2^ ^,^ ^5^ ^,^ ^13^ ^,^ ^15^ ^,^ ^33^. Particularly inspiring was the rereading of *Pedagogy of Hope: Reliving Pedagogy of the Oppressed* ^34^. Freire discusses how disaster and emergency situations can paralyze action, but insists that such situations (like the deadly droughts and floods historically faced by Brazilians) also mobilize the collective and solidarity-based innovation needed to confront them, prompting the building of what he calls “*untested feasibility*”: innovating requires stepping out of the comfort zone of known practices in order to “*collectively reinvent*”.

We were “*navigating a terrain that moves and shifts even as we attempt to traverse it*”, as Burawoy ^35^ (p. 19) would say, and the ethnography that described previously unexperienced daily lives and settings remained online until October 2021, when vaccination reached the school communities.

During this period, we designed approaches historically conceived as psychosocial, psycho-educational and dramaturgical, as opposed to behavior-modeling socio-psychological approaches, moving beyond the prevention education that Freire would describe as “*banking education*” ^5^ ^,^ ^36^. Based on the decoding of everyday life carried out in online sessions with the youth, we developed descriptions of scenes of exposure to different adversities (COVID-19, STIs, unplanned pregnancy, violence and psychosocial distress) experienced or observed by the student youth agents. We then expanded them to understand how they reflected the shared context that structures the practices of each individual’s personal trajectory and wishes. Supporting the participating adolescents and youth, we experimented with reenacting the scenes through guided imagination ^37^, aiming at untested viability so that future interactions could be safer. The unit for decoding and intervention was the youth themselves, conceived as rights-bearing individuals within scenes of their daily lives ^5^ ^,^ ^15^ ^,^ ^33^.

In the initial months of our video-ethnography, we focused on building an organic connection with the youth, teachers and school leadership to monitor their initiatives and challenges in understanding and promoting prevention within each territory, as well as in seeking solid evidence on COVID-19 transmission and prevention methods. During this time, the youth agents observed drastic changes in their households and, through TV and digital platforms, witnessed a world brought to a standstill, paralyzed by fear. They described and discussed specific experiences in their school communities and neighborhoods - isolated within the territories where they lived ^26^ ^,^ ^38^. Girls often “*observed from the window*” or heard stories from those who ventured outside. Most of the boys went out into streets dominated by men, a form of masculinity encouraged by President Bolsonaro. Free from domestic chores and child care - responsibilities that burdened the girls - they would tire of playing video games, seek out soccer games or be tasked by their families with finding work to replace unemployed adults. In conversations, girls questioned them for breaking social isolation and exposing vulnerable family members to risk. However, the boys justified this by saying that the people they met were “*acquaintances*”, thus ignoring the risk of infection by or transmission of SARS-CoV-2 when meeting friends. In other words, “*the risk was reduced*” if the person was “*known*”, as in the AIDS epidemic.

**Figure 1:**
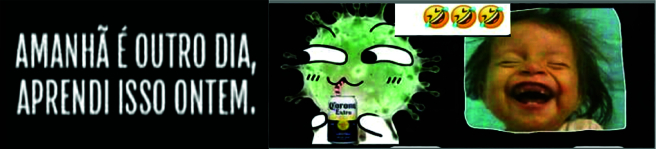
On WhatsApp, a boy expresses his feelings about leaving “quarantine” to go to work.

Note: illustration taken from a WhatsApp group, with no identified author, no title and no date.

During the period with no in-person classes or vaccines, we held “*synchronous*” discussion circles on meeting platforms, “*asynchronous*” conversations on WhatsApp and online focus groups on topics that frequently emerged across different territories (Figure 1). The online interactions with youth agents made it possible to decode significant scenes they experienced or observed, prompting reflection on issues related to distress in everyday life, such as gender inequality in domestic work and employment, and “harassment”, “racism” and “police violence”. The rationale for breaking “quarantine”, as they called it, was complex and dynamic, often accompanied by nervous laughter.

Concurrently with the US-based #MeToo movement, we observed an increase in reports of harassment in schools by teachers, staff and peers ^39^. Physical distancing did not prevent the persistence of such incidents. We discussed cases such as the teacher who masturbated during an online class and unknown men who infiltrated virtual classrooms to perform obscene gestures and expose themselves on camera.

In this context, dominated by far-right politics, police violence increased dramatically in peripheral areas, prompting the Brazilian Supreme Federal Court to limit police incursions in *favelas* during the pandemic ^40^. Meanwhile, in the online discussion circles, police violence sparked debates about racial oppression, which evolved into conversations about discrimination observed first-hand by youth. They discussed the school environment, where black teachers were a minority and black staff were confined to cleaning and technical support duties. The “structural racism” addressed by “influencers” started raising awareness of the lack of educational and employment opportunities.

The experiences of racism, harassment and gender-based violence, combined with grief - 30% of respondents in 2022 had lost neighbors or relatives to COVID-19 - highlighted the need to address the significant increase in mental distress we had already identified in 2019 ^26^. This reinforced our understanding of the need to provide support for what we identified as psychosocial distress, deeply rooted in the discrimination and violence associated with racism, sexism and LGBTQIA+ phobia ^41^ ^,^ ^42^.

**Figure 2:**
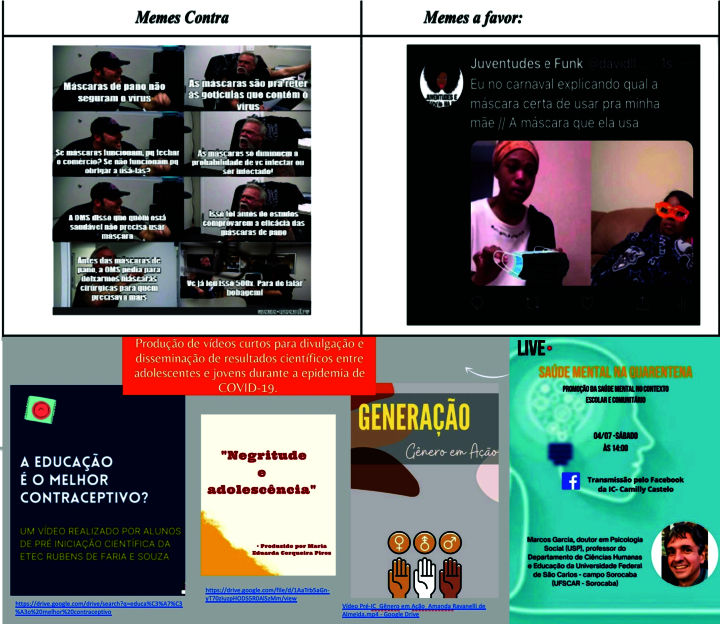
Memes, videos, and posters sharing activities created by youth.

Note: illustrations taken from a WhatsApp group, with no identified author, no title and no date.

Through weekly meetings via cell phones and screens, we supervised the prevention youth agents. Step by step, we assessed the process of producing and implementing “*online COVID-19 prevention workshops*”, monitoring the initiatives of adolescents and youth, who produced highly creative materials about the importance of staying home, wearing face masks and getting vaccinated. Over 18 months, they produced messages, memes, small-scale research, videos, thematic live streams and posters shared via WhatsApp, Instagram, Facebook and YouTube (Figure 2). This sustained dialogue allowed the youth agents to engage with their peers and what they defined as their “audience”: schoolmates, friends and also relatives and neighbors.

Sexual orientation and gender identity were “hot” topics when we discussed the questionnaire results - administered in 2019, debated online in 2020, and administered in 2022 - which confirmed that a significant percentage of youth self-identified as non-heterosexual, transgender, nonbinary or questioning. Family conservatism was cited as a major source of distress for LGBTQIA+ youth at home and a strong factor in “*breaking quarantine*” (going out in defiance of physical distancing recommendations).

Regarding discrimination at school (which they referred to as “*bullying*”), they stressed that the “*playful tone*” of such behavior made it more difficult to report and resist rights violations related to racism and the process of embracing non-traditional sexual and gender identities, an experience deeply ingrained in their school life. They emphasized that this form of discrimination causes distress and must be addressed as “*a serious problem*”.

Discussing sexuality and preventive practices online was challenging, even after the youth agents had learned to protect their privacy by turning off cameras and/or audio at home. When we finally revisited the topic of sexuality with the return to in-person schooling in late 2021, the students were relieved: “*Phew, enough of COVID!*”.

However, discussions about “sex during the pandemic” and interesting and acceptable prevention practices were met with strangeness rather than discomfort. Protected sex sounded bizarre: wearing a face mask during sexual intercourse? This subject made them laugh, especially in relation to the questions in the 2022 survey about the preventive measures used during the first and last time they had sex. Answers included “*COVID-19 testing before sex*” and “*showering and personal hygiene before sex*” - a practice traditionally associated with “after sex” and largely ineffective for preventing STIs. Safe sex was only at home with casual partners or boyfriends/girlfriends who had become “exclusive” during the early months of COVID-19, whether in the absence of parents or with their consent. Using condoms or birth control pills? “*They used to give them for free at the health center... do they still? It’s packed with COVID patients!*”.

In person, we conducted focus groups on the impact of COVID-19 on their lives, during which they described their exclusively online existence with phrases like: “*in the times of the zombie apocalypse*”. They referred strongly to the fatigue and difficulty of permanently adhering to COVID-19 prevention measures still in place, highlighting the challenges of simultaneously preventing harms resulting from other ongoing pandemics - such as syphilis and HIV - and the psychosocial distress already viewed by the school community as “pandemic”.

“*When I caught depression*” was the expression used by adolescents and youth to convey their feelings during this syndemic period, which enhanced their understanding of the epidemic nature of mental and psychological distress, described as if it were a viral event, spreading in a similar manner. We continued documenting the overwhelming sensations of what students identified as “anxiety” and cumulative, intensified fears ^38^ ^,^ ^42^, interviewing teachers close to them who reported being “unprepared to address the issue” and often experiencing similar distress.

All the fieldwork conducted with and by youth agents - including discussion groups on lived or observed experiences, workshops, focus groups and participant observation of life in the neighborhood or in different school settings upon the return to in-person classes, as well as the data collected from the questionnaires and the debates about their results - indicated that friends and peers were the most important and effective source of support to face what the media was already referring to as an “epidemic of mental disorders”, viewed by us from a psychosocial perspective in interaction with mental health frameworks based on human rights ^43^.

As we have discussed in other papers ^41^ ^,^ ^42^, the questionnaires revealed rates of psychological discomfort (which we view as psychosocial distress) above the global average. Mental healthcare was the top priority chosen by students when asked about the most urgent needs and demands for health centers in the territory - though they criticized the specialized mental health centers for their long waiting lists and group-focused care, which often did not meet their expectations.

## From combined prevention to comprehensive prevention

Faced with the emergence of COVID-19 and inspired and challenged by the memory of the response to AIDS, we promoted social distancing, COVID-19 testing and the proper use of face masks as measures to prevent transmission during inevitable or desired gatherings. In 2021, we encouraged vaccination efforts. By 2022, during in-person workshops on pregnancy and STIs (syphilis and AIDS) prevention, mpox emerged, particularly alarming LGBTQIA+ youth. At that time, we investigated in greater detail the increase in psychosocial distress we had already observed in 2019 ^26^. By 2023-2024, schools were once again grappling with dengue fever outbreaks.

In this syndemic context, as we strove for innovation in the unstable ground we were traversing, the importance of a comprehensive prevention approach became evident. Amid the polysemic and polymorphic ways in which this principle of SUS has been incorporated ^44^, we adopted a comprehensive framework based on the theory of healthcare work process ^45^ and its related concept of healthcare ^46^. This framework focuses on four core areas for (re)constructing health practices: (i) addressing health needs beyond strictly biomedical demands, adopting a holistic approach where health initiatives offer meaningful responses; (ii) integrating health promotion, disease prevention, treatment and rehabilitation into the objectives of health initiatives; (iii) establishing interdisciplinary and intersectoral collaboration to better achieve healthcare goals; (iv) facilitating dialogic, plural and constructive interactions among the various stakeholders involved in health initiatives ^47^.

Despite being widely discussed in the context of organizing healthcare within the SUS, little reflection has been given to implementing the principle of comprehensive care in the field of prevention. Prevention initiatives have often followed a verticalized approach, focusing on specific health issues without considering the social and programmatic contexts that contribute to the vulnerability of individuals and groups, and ignoring other coexisting health issues and demands present in the same everyday scenes.

Adopting the term “comprehensive prevention” reflects efforts to better manage the synergies between adversities, understand the redefined needs and demands in their broader contexts, and reconstruct daily approaches to address them. In other words, the goal of engaging with the three analytical dimensions of vulnerability (personal, social and programmatic) and the human rights framework is not to juxtapose or prioritize needs and demands but to activate responsive capacities in ways that align with the practical significance and mutual interplay of these needs in the daily life of adolescents and youth.

In this sense, we aimed to dynamically and comprehensively address events that overlapped synergistically - COVID-19, STIs/AIDS, unwanted pregnancy, violence and psychosocial distress. In 2022, when we were finally able to meet in person, we implemented this preventive educational approach in what we called the “Comprehensive Prevention Workshop”. Planned as a six-session program, the workshop aimed to decode real-life interaction scenes where we discussed when and how to use face masks, condoms, contraceptives, emergency contraception, PEP (post-exposure prophylaxis) and PrEP. The youth agents used salt dough to model sexual and reproductive anatomy, practiced applying and removing male condoms on a cucumber/carrot, and inserted internal condoms in 3D models. They also mapped high-exposure sites in their territories and identified how to access prevention supplies, HIV and COVID-19 self-tests, and vaccinations for COVID-19 and HPV. During these sessions, they imagined their own ways of embodying prevention and seeking support for psychological discomfort.

Using scenes as units of interpretation to understand vulnerability to adversities that syndemically endanger the physical and mental health of adolescents and youth, and which we considered to be densely embedded in their everyday lives, proved to be a productive approach. It helped us to problematize social vulnerability and, through interventions, to understand the mutual interaction between health issues and the practical, desirable responses to them.

**Figure 3:**
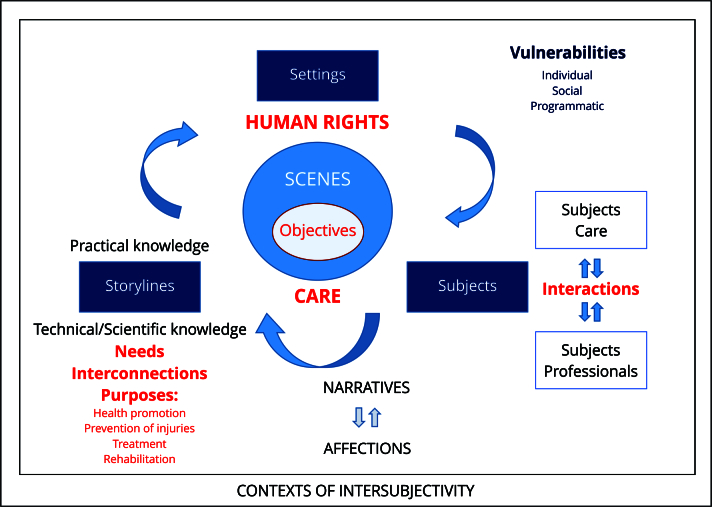
Care, comprehensiveness, vulnerability and human rights.

Reconstructing vulnerability scenes through the lens of healthcare guided by the protection and promotion of rights brings visibility to individuals, contexts and storylines that help contextualize potential objectives for practical health promotion and prevention initiatives. Thus, by focusing on intersubjective contexts shaped by structural determinants (material conditions, power relations, cultures), as well as the affects and narratives that connect us to them, we became better equipped to foster dialogue between technical/scientific and practical knowledge, aiming for effective comprehensive prevention, as illustrated in Figure 3.

We progressed from the proposal of combined prevention for STIs/AIDS we had been working to implement, since, once again, we were challenged by the interconnected and underlying aspects of offering preventive resources that only appeared to be equitable and equivalent in their practical implications.

The combined prevention proposal emerged within a context of political regression observed in the response to AIDS in Brazil and worldwide, when discussing sexuality or engaging with individuals about their sexual experiences on their own terms shifted away from the increasingly biomedicalized focus of prevention. “Combined prevention” ^48^ ^,^ ^49^ starts as a well-intentioned attempt to link biomedical prevention methods with the “behavioral” prevention tradition, which we prefer to call “psycho-educational” or “psychosocial approach” ^5^ ^,^ ^36^. It takes into account power and cultural differences, emphasizing the socio-structural determinants of health and illness ^31^. However, its implementation, much like the approach of the Brazilian government at the time, began to avoid confronting the conservative and religious discourse that restricts human rights-based sexuality education, especially for children and adolescents. Many professionals in education and healthcare, and even adolescents and youth, found it easier to avoid such confrontation and replace the conversation with “prescribed pills” for prevention.

Without disregarding the importance of having a wide range of preventive resources available within the SUS, we cannot ignore the historical, economic, political and cultural contexts in which healthcare technologies, including preventive ones, are produced ^50^. This requires being alert not only to the different possibilities and interests in accessing various prevention methods and resources in each particular context, but also to the ideological and political implications of each of these options. It is especially important to highlight the risk of adopting, often uncritically and inadvertently, the individualistic approach of “fractional universalization”, i.e., the ideologically biased conception that the goal is to offer everyone the available prevention resources, whose use and results will, however, be the exclusive responsibility of each individual ^23^.

Throughout the project, as we developed the comprehensive prevention approach, we aimed to co-construct transversal skills for the prevention and promotion of comprehensive health - of the youth and of their peers and friends - through psychosocial interactions in the community. The goals were:

(a) to dispel the idea of the inevitability of health issues and mobilize students as rights-bearing individuals - to education that nurtured their future study and work plans; to comprehensive health; to quality food and housing; to non-discrimination based on color, gender, sexual orientation, physical characteristics, etc.;

(b) to have access to the best technical-scientific consensus, to learn how to keep up to date - about prevention measures and available resources - and to “embody” such knowledge in everyday scenes of varying exposure to ongoing pandemics;

(c) to understand that experiences change according to each everyday scene and its context, being alert to decode each psychosocial interaction scene.

In dealing with the pandemic of mental health issues, we maintained the human rights-based perspective emphasized by international organizations ^43^ and began to face the challenge of thinking about the prevention of psychosocial distress also as a collective task - of schools and community projects dedicated to adolescents and youth - criticizing its excessive medicalization and individualization.

In the almost weekly meetings with students, after vaccination and the return to in-person classes, the experience with COVID-19 was summarized in sentences such as: “*The pandemic is over, but discrimination and violence never end*”. These statements reflected an interpretation of reality by those who live it, and in interaction with the research team, who helped decode their scenes and contexts and promote prevention, they built knowledge committed to equality and justice.

Meetings with teachers and family members to discuss results complemented the interpretations and information about lived experiences, helping sustain the project’s feasibility. It was significant to observe that many parents and educators, when they were adolescents and high school students, had attended human rights-based AIDS prevention and sexual education programs at school, such as “Prevention is Also Taught” ^51^. We confirmed how, even in contexts of regression and neglect, memories of responses to a public health crisis like AIDS combine community and professional experiences, pointing to pathways for promoting health in a territory.

In other words, we ensured successful experiences in responding to the synergy between health risks and in promoting comprehensive prevention to mitigate vulnerabilities, valuing participation (an organizing principle of the SUS) as the coordinating principle of health initiatives based on human rights, such as non-discrimination, accessibility, availability and acceptability of healthcare services, and of social practices when people received information in appropriate language based on technical and scientific evidence to make well-informed decisions, respecting their privacy and confidentiality ^15^ ^,^ ^26^.

In this syndemic context triggered by COVID-19 in 2020, we reaffirmed the productivity of the human rights framework in anticipating and mitigating vulnerability in socially marginalized territories, as COVID-19 would never be restricted to the west region of São Paulo, where middle-class tourists who had been to Italy and other European countries were arriving, bringing the virus. We confirmed the adage that the greater the inequality, the greater the violation of rights and the vulnerability to infection, illness and psychosocial distress, which underpinned the prevention discussion and training sessions for professionals and the segments and territories we have been addressing in this context for two decades.

## Conclusions

This project is among those that supported a Brazilian social response to COVID-19 based on human rights, opposing as best as possible the evidently tragic outcome - still not fully calculated due to the undoing of the historical tasks of the Brazilian Ministry of Health between 2018-2022 - of the violation of fundamental rights and of the denialist and negligent policies implemented by the Federal Government during the four years it was led by politicians who openly disregard human rights. Despite the election of increasingly conservative governments, the collaboration between academia and civil society in the response to the syndemic exacerbated by COVID-19 held administrators accountable and validated programmatic initiatives to oppose inequities structured in gender, class and race relations.

A key aspect of this model of comprehensive prevention is focusing on understanding resistance to local inequality and inequity, enhanced by community participation in the territory and capacity-building. The prevention youth agents we worked with faced the polarization and aggressiveness of the far-right and conservative sectors driven by moral panic, which hindered intersectional, human rights-based responses to the syndemic. Thus, we learned that public discussions on how to prevent violence, abuse and sexual harassment facilitate interaction with conservatives who do not fully agree with the far-right sectors leading the “moral panic”, including segments of the neopentecostal churches active in the territories. When translated into the language of human rights violations, these issues prioritized by conservatives helped to understand the inseparable relationship between greater or lesser vulnerability and the disrespect or protection of human rights, increasing the possibility of community collaboration within this framework.

Online life not only required collective creativity in unprecedented and feasible methodologies but also stimulated the creativity of this generation of adolescents and youth, who were born in the internet era, to co-produce prevention materials basically shared on Instagram and WhatsApp. It once again highlighted the need to actively seek practical knowledge, such as the everyday experiences of adolescents and youth in navigating themselves, their relationships and their context (which the scene method supports), so that the necessary technical and scientific knowledge on prevention may complement rather than replace or ignore it. Without this combination ^52^, our prevention knowledge and proposals make no sense, and the practical situations we aim to address (relationships, narratives, affects) will remain opaque to us.

Therefore, we learned that - especially in times dominated by cell phones, apps and social media - conservative efforts are unable to obstruct the debate on sexualities and genders (identities and practices) and eradicate the youth experience driven by the right to non-discrimination of bodies, identities and desires that are undervalued and stigmatized ^53^.

Unlike our experience in responding to AIDS, which was strongly supported by identity movements, the academia-civil society collaboration was largely organized around communities defined by their territories, a lesson that must not go to waste ^54^. Despite being limited by the political context, the collaboration and creativity of the community supported the recognition of adolescents and youth as rights holders - to health, to prevention in schools and to non-discrimination - without allowing their differences (of religion, ethnicity, sexual orientation, gender) to result in discrimination and exclusion.

When invited to co-create a scene of exposure to COVID-19, the youth incorporated “consent” into interactions and physical proximity, and learned to recognize prevention resources (face masks, vaccination, testing) and how to access them (and the obstacles to doing so). They increased the likelihood of doing the same for scenes of exposure to sexually transmitted infections and unwanted events like pregnancy and intimate partner violence. By being able to decode the scenes and settings in which they are vulnerable, they develop greater autonomy and produce social solidarity, while identifying how, where and when to access and use (or not) the various resources and strategies for comprehensive prevention.

In line with the above, our thesis is that the comprehensive prevention resources outlined throughout this project, and which we continue to evaluate, enable individuals to recognize both themselves and others as rights holders who are inherently connected to the groups and contexts in which they live. Fostering comprehensive prevention contributes to understanding the simultaneity of vulnerability factors within those contexts and how the resources must be integrated to produce effective responses.

However, as stressed by the students, who included addressing their psychosocial distress as a priority in the ongoing syndemic of pandemics, it will be complex and difficult to think and act “*facing and preventing everything at the same time*”.

In the territories where we worked, we observed that, with each everyday scene and each new disaster, individuals, groups and communities end up choosing which “threads to pull” from the fabric of comprehensive prevention and care. In other words, with each accumulating emergency and each disruption caused by environmental disaster, priorities emerge as each one pulls the thread that feels most urgent for them and/or their territory. It might be hunger, lack of housing destroyed by fire or flooding, the pursuit of pleasurable sex free from disease and psychosocial distress or access to vaccination. In prevention literacy, we insist that when pulling the thread, we should not lose sight of the ball of indivisible rights that guarantees greater dignity for our shared humanity - the right to food, to decent housing and work, to quality healthcare, from prevention to rehabilitation.

Lastly, with COVID-19 and dengue fever in 2023/2024, we shifted our understanding of the severity of the effects of the ongoing environmental crisis, especially on health. If it astonishes us that “*the world hasn’t changed!*” as much as the youth and we had hoped, we cannot go back to what we did before without advancing and reformulating. To change the world, even while traversing uncertain ground, prevention professionals must actively and critically engage in the debate on preparedness for the next and new pandemics. Unbelievable as it may seem, community participation in each territory and theoretical and methodological production on prevention are still absent from this debate.

The syndemic nature of health emergencies forecast in times of climate and environmental crisis, and the preparedness required to face them, have expanded the challenges of sustaining prevention and health promotion programs in schools. In this sense, we stress that schools are always a point of reference in their territory and can mitigate vulnerability to unnecessary and preventable suffering, especially for children and youth. In other words, focusing on research and social technologies that guarantee the right to prevention and participation, starting in the school territory, should be part of preparedness for health crises. We must produce literacy to “decode” everyday scenes of social and sexual interactions in the territories where we live and circulate, which will allow us to anticipate exposure and stimulate imagination about how to “embody” prevention - of unplanned pregnancy, harassment and sexual abuse, and, at the same time, of infectious diseases like STIs/AIDS, COVID-19, mpox, TB, dengue fever and the w, x, y, z that will come with droughts, floods, landslides, interactions between different biomes and the rapid movement of people across continents.

Even in adverse contexts, we continue to uphold the productivity of human rights principles and their indivisibility, as well as of comprehensive healthcare and prevention strategies that reduce vulnerabilities, key aspects to respond to these times of a “zombie apocalypse”.
