# Supplementary material for: From “combined prevention” to “comprehensive prevention”: building the response to the syndemic with adolescents and youth in São Paulo, Brazil (2020-2023)
Source: Cad Saude Publica. 2025 May 19;41(4):e00084323. doi: 10.1590/0102-311XEN084323 (PMC12091862; doi:10.1590/0102-311XEN084323)
Supplement: Translation

# Translation: Da “prevenção combinada” à “prevenção integral”: construindo a resposta à sindemia com adolescentes e jovens em São Paulo, Brasil (2020-2023)

**DOI:** 10.1590/0102-311XPT084323

**Authors:** Vera Paiva, José Ricardo de C. M. Ayres, Ivan França, Marcos Roberto Vieira Garcia, Cristiane Gonçalves da Silva, Júlio Assis Simões, Luis Guilherme Galeao-Silva, Jan Billand, Grupo de Pesquisa sobre Saúde dos Jovens e Direitos Humanos

**Affiliations:** 1 Instituto de Psicologia, Universidade de São Paulo, São Paulo, Brasil.; 2 Faculdade de Medicina, Universidade de São Paulo, São Paulo, Brasil.; 3 Faculdade de Saúde Pública, Universidade de São Paulo, São Paulo, Brasil.; 4 Universidade Federal de São Carlos, Sorocaba, Brasil.; 5 Universidade Federal de São Paulo, Santos, Brasil.; 6 Faculdade de Filosofia, Letras e Ciências Humanas, Universidade de São Paulo, São Paulo, Brasil.

**Keywords:** Adolescente, Prevenção de Doenças, Integralidade em Saúde, COVID-19, HIV

## Abstract

Este ensaio discute a necessidade, e possibilidades, de transitar da orientação conceitual da prevenção combinada para a concepção de “prevenção integral”, com base em experiências de pesquisa-intervenção em promoção e proteção da saúde sexual e reprodutiva entre adolescentes e jovens de três cidades do Estado de São Paulo, Brasil (São Paulo, Santos e Sorocaba). Potente na preparação para ciclos sindêmicos no contexto de crise ambiental, a concepção de prevenção integral favoreceu a inteligibilidade de processos de vulnerabilização à COVID-19 e à mpox, às infecções sexualmente transmissíveis e à aids, à violência, à gravidez indesejada e ao sofrimento psicossocial. Permitiu orientar respostas à sindemia de pandemias em meio à crise social que as agrava e estimulou a construção pelos jovens de respostas criativas diante ao contexto de polarização política promovida pela extrema direita - que agressivamente inibe abordagens baseadas em direitos humanos, em especial no campo da sexualidade, agudizando a infodemia que desqualifica a gravidade das pandemias em curso. Ressaltamos a produtividade da concepção de integralidade do cuidado para delinear ações no quadro dos direitos humanos e prevenção que, dinamicamente, antecipam e abordam eventos que se sobrepõem sinergicamente em territórios periféricos. Ao valorizar “cenas” como unidade interpretativa e foco das respostas a contextos de vulnerabilização, favorecemos a coconstrução de competências transversais nas respostas pessoais, coletivas e territoriais necessárias à prevenção de agravos que concomitantemente ameaçam a saúde física e mental de adolescentes e jovens.

## Introdução

Desde os anos 1990, em diferentes projetos de extensão e pesquisa no campo da sexualidade, promoção de direitos da juventude e prevenção da aids ^1^ ^,^ ^2^ ^,^ ^3^ ^,^ ^4^ ^,^ ^5^ ^,^ ^6^ ^,^ ^7^, celebrávamos o prazer - e não apenas os riscos -, a diversidade sexual e de gênero e diferentes culturas religiosas, garantidas na laicidade do Estado pela *Constituição Federal* promulgada na época. No Estado de São Paulo, além do interesse por infecções sexualmente transmissíveis (IST)/aids, respondíamos ao ininterrupto interesse de adultos e jovens na prevenção da gravidez.

Na virada do século XXI, nos territórios em que permanecíamos promovendo saúde sexual e reprodutiva, trabalhamos com adolescentes e jovens crescendo em famílias que se filiavam a igrejas cristãs evangélicas, parte delas recém-convertidas e dedicadas à pregação de seus valores e moral sexual e, pouco comum desde a redemocratização, à “demonização” de outras tradições religiosas. Baseados no quadro da vulnerabilidade e direitos humanos ^8^ ^,^ ^9^, investigamos as religiões no âmbito da resposta à aids e acompanhamos desde 2010 o retrocesso em políticas públicas defendido por políticos que usavam o discurso religioso fundamentalista, evangélico e católico, para defender “*a natureza do corpo*” e denunciar o que chamaram de “*ideologia de gênero*” ^10^ ^,^ ^11^ ^,^ ^12^ ^,^ ^13^.

A cada investimento em pesquisa-intervenção, mostrávamos como os territórios onde adolescentes e jovens circulam, moram e estudam definem singularidades inescapáveis para compreender e mitigar a vulnerabilidade ao adoecimento ^6^ ^,^ ^7^ ^,^ ^14^ ^,^ ^15^. Territórios têm história e cultura e, nas periferias, definem comunidades com base no compartilhamento de visões de mundo e no processo de resistência política à exclusão, à estigmatização, à violência baseada na sexualidade e no gênero e ao mal-estar psicológico. A conexão territorial orienta a instalação das unidades de serviços públicos do Sistema Único de Saúde (SUS) e do Sistema Único de Assistência Social (SUAS) seguindo princípios de regionalização e descentralização. A lógica da conexão territorial também orienta a localização das igrejas cristãs, como observamos, em especial as neopentecostais.

A ação social e política nos territórios modula as possibilidades de manutenção da saúde da comunidade, as oportunidades para mitigar vulnerabilidades ao adoecimento em um campo de tensões entre dominação (seu uso para o lucro-troca) e apropriação (para sobrevivência, produção social e cultural) ^16^.

A eleição de Jair Bolsonaro à presidência (2018) e, desde então, de governos estaduais abertamente conservadores de extrema direita aceleraram os retrocessos. Entre 2010 e 2021, sem acesso à educação sexual e prevenção compreensiva baseada em direitos humanos, a vulnerabilidade de adolescentes e jovens cresceu: a taxa de detecção de casos de aids (por 100 mil habitantes) na faixa etária de 15 a 24 anos variou de 11,5 a 13,3, enquanto diminuía o uso de preservativos, sem que aumentasse o número de jovens e adolescentes entre os usuários de profilaxia pré-exposição ao HIV (PrEP) no SUS ^17^.

Em 2020, quando o mundo parou diante da gravidade da pandemia da COVID-19, formávamos estudantes de Ensino Médio como agentes-jovens de pesquisa e prevenção de IST/aids e gravidez, em metodologias de perspectiva interseccional, e sobre o impacto da estigmatização e da discriminação de direitos no âmbito de uma pesquisa-intervenção (*Vulnerabilidades de Jovens às IST/HIV e à Violência entre Parceiros: Avaliação de Intervenções Psicossociais Baseadas nos Direitos Humanos*) ^18^.

Desenvolvemos o projeto em um contexto desafiador, respondendo à dinâmica de duas crises simultâneas:

(1) Desdobramento da sindemia de doenças infecciosas (COVID-19, IST - sífilis e aids -, mpox, dengue) e de sofrimento psicossocial no contexto de crise sociopolítica que as agrava. Como definiram Singer et al. ^19^ uma abordagem sindêmica examina os caminhos que produzem a sinergia entre doenças, multiplicando danos, e como a desigualdade e a injustiça contribuem para sua interação e para a vulnerabilidade social. Em 2020, quando Horton ^20^ definiu o caráter sindêmico da COVID-19, sublinhou a necessária resposta multisetorial em ações na saúde e na educação, em políticas de moradia, emprego, alimentação, meio ambiente, respostas que deveriam ser articuladas especialmente desde o plano “*local*”, que concebemos como “*territorial*”.

(2) Polarização política, a estratégia de tensionamento político agressivo da extrema direita que polariza tudo e todos para distribuir rumores e desinformação amplificadas pelas redes sociais. Esse tensionamento político produz “*anti-agendas*” conservadoras nos campos da aids ^21^ e da saúde reprodutiva que inibem abordagens no quadro de direitos humanos e, indo além, agudizam a infodemia que sustenta a manipulação de informações com intenção bloquear o acesso e a validação de fontes e orientações confiáveis sobre como se prevenir.

Buscávamos implementar e avaliar um modelo de prevenção combinada de HIV/aids em oito escolas de Ensino Médio de forma integrada a unidades de saúde no mesmo território, nas cidades de São Paulo, Santos e Sorocaba. Almejávamos, portanto, a “*redução de novas infecções pelo HIV ao combinar estratégias biomédicas, comportamentais e estruturais baseadas nos direitos humanos e em evidências, considerando contextos locais*” ^22^.

Entre as diversas lições aprendidas nessa experiência, no período de 2019 a 2023, ressaltaremos neste ensaio os giros conceituais reclamados pela necessidade de superar a “*universalização fracionária*” ^23^ das propostas mais adotadas em prevenção, mesmo na perspectiva da prevenção combinada. A simples oferta de uma “*cesta*” de opções preventivas a serem “*consumidas*” ^5^ segundo a conveniência de cada pessoa, nos moldes de certa ideologia neoliberal ^24^, parece-nos insuficiente, senão um obstáculo mesmo, para a construção de práticas efetivas de promoção da saúde e para uma preparação adequada para responder aos novos ciclos sindêmicos que deveremos continuar enfrentando nos próximos anos.

O objetivo deste artigo é discutir a necessidade, e apontar possibilidades, de transitar da orientação conceitual da prevenção combinada para a concepção de prevenção integral no desenvolvimento de ações de promoção e proteção da saúde sexual e reprodutiva em contextos sindêmicos, tomando centralmente como base essa experiência de pesquisa-intervenção de base territorial desenvolvida pelos autores.

## Adaptando metodologias de pesquisa e intervenção “no tempo do apocalipse zumbi”

Atravessada pela COVID-19, a pesquisa-intervenção que inspira este ensaio exigiu refinamento no processo de consentimentos permanentes da comunidade escolar nos oito bairros periféricos e favelas eleitas para o estudo e, a cada mudança no contexto e desenho, aprovação do Comitê de Ética em Pesquisa com Seres Humanos do Instituto de Psicologia, Universidade de São Paulo (USP; protocolos: nº 2.979.702 em 24 de outubro de 2018, nº 4.079.347 em 9 de junho de 2020 e nº 5.334.405 em 6 de abril de 2022). Depois do consentimento institucional nas três cidades, o primeiro passo foi convidar alunos das escolas técnicas e regulares de Ensino Médio para participar do projeto.

Não observamos diferença significativa no perfil dos estudantes participantes no período. Em 2019, 719 estudantes do 3º ano responderam a perguntas que buscavam identificar a sua vulnerabilidade (pessoal, programática e social) às ISTs, à violência entre parceiros e pares, e ao sofrimento psicossocial. Acessaram o questionário usando links em *tablets* e computadores na escola. Em 2020-2021, em parceria com os agentes-jovens e professores com quem seguimos interagindo online, respondemos à emergência da COVID-19, desenhamos e implementamos atividades de pesquisa e prevenção no território acompanhadas de registro etnográfico, que instruíram a reconstrução do questionário e seus modos de abordagem. Em 2022, 1.242 estudantes de 3º ano que se formavam após terem cursado o Ensino Médio nos anos mais agudos da COVID-19 responderam presencialmente ao questionário que incluiu perguntas sobre essa experiência. A maioria identificou-se como branco(a) e amarelo(a) (52%), 48% preto(a), pardo(a) ou indígena. Manteve-se alta a proporção dos “sem religião” (44%) e a proporção de católicos (20%), evangélicos-pentecostais (17%), de umbanda/candomblé (5%) e dos que indicaram outras afiliações religiosas ou não quiseram responder (5%). A proporção dos que se identificavam como homens permaneceu cerca de 44%, 52% como mulheres e 5% não se classificaram como “homem” ou “mulher”, escolhendo a alternativa “me identifico de outra forma”. Identificaram-se “como gay/lésbica/pansexual/outra forma/em dúvida” 20% dos rapazes e 45% das moças.

A equipe de pesquisa - interprofissional, interdisciplinar e intergeracional - que incluiu 240 estudantes de Ensino Médio formados como agentes-jovens de 2020 a 2023, seguiu lidando com o contexto, almejando informar políticas públicas baseadas em direitos humanos, reconhecendo que adolescentes e jovens têm classe, raça/cor, gênero, sexualidades e religiosidades diversas, diferenças que estruturam inequidades.

Desde o estudo preliminar, cujos resultados foram discutidos em encontros e rodas de conversa online com os agentes-jovens e professores dos oito territórios escolares no projeto, já identificamos os efeitos do retrocesso na promoção e proteção da saúde sexual resultante da ascensão de governos conservadores e abandono neoliberal das políticas sociais ^24^. Como em outros estudos brasileiros sobre prevenção combinada entre jovens ^25^, observamos como a vulnerabilidade social ao adoecimento expressava o cotidiano nos territórios onde jovens circulam, marcadas(os/es) pela iniquidade.

A polarização política, mais intensa nos períodos eleitorais, mobilizou pais e mães contrários ao projeto e professores conservadores, frequentemente silenciando profissionais da educação que temiam uma reação agressiva contra a escola; manifestava-se nos comentários provocativos nos debates da apresentação dos resultados dos questionários e na resistência tímida de professores e dirigentes escolares à surpreendente adesão ao negacionismo de colegas que ensinavam ciência. Mesmo assim, a criatividade de gestores escolares e estudantes no esforço para sustentar a pesquisa-intervenção permitiu contornarmos o impacto e persistente legado da pandemia, que segue afetando desproporcionalmente uma geração de adolescentes e jovens ^26^ ^,^ ^27^ ^,^ ^28^.

A agressiva atuação da extrema direita que favoreceu a disseminação do vírus está fartamente documentada e analisada ^29^ ^,^ ^30^. Especialmente no período discutido neste artigo, a falta de liderança e de coordenação entre os governos federal, estadual e municipal deixou uma conta histórica para adolescentes e jovens que nomeamos de impacto longo da COVID-19 - que inclui o impacto psicossocial que não cabe nas definições correntes de COVID-longa (restritas a eventos biomédicos).

Diferenças nas respostas às epidemias de aids e COVID-19 são notáveis. As condições adversas e complexas na emergência da COVID-19 - uma doença transmitida pelo ar e não apenas com trocas de fluidos corporais, como as ISTs - e o grande impacto da morbimortalidade exigiam uma escala muito maior de respostas. Ao mesmo tempo, na construção da resposta brasileira à aids, vivíamos o final de ditadura e a derrota da extrema direita violenta que a sustentou, em um processo de democratização crescente com a colaboração entusiasmada de gerações que, em vários setores da vida social, dedicaram-se à construção de um Estado de bem-estar social e de políticas baseadas em direitos humanos.

Guardávamos como legado da resposta à aids o consenso sobre a determinação social e estrutural das pandemias e sobre a importância da organização comunitária na produção de respostas efetivas para seu controle. Parker & Camargo Jr. ^31^ já ressaltavam o efeito sinérgico da determinação estrutural e da exclusão social na infecção pelo HIV e no adoecimento por aids, ao discutirem “*a sinergia de pragas*” e a “*aids como sindemia*”, nos termos de Singer.

No plano da resposta psicossocial implicada nas ações de prevenção e da experiência individual, também aprendemos com a aids que: (a) o medo pode aprofundar a negação do risco e a estigmatização de grupos de risco; (b) a solidariedade social é crucial para enfrentar as epidemias locais; (c) a mudança de comportamentos e das práticas de interação social seria sempre uma tarefa complexa e multifacetada, que dependeria de tempo para que as pessoas incluíssem a experiência viva do autocuidado e cuidado dos outros, avaliada e modificada na dinâmica de interação em cada situação de vulnerabilidade.

Na resposta à COVID-19, por outro lado, o lugar dos jovens nas avaliações de vulnerabilidade mudava de “*grupo de maior risco*” na pandemia de HIV/aids para grupo menos suscetível (que os mais velhos), argumento usado reiteradamente por governos e ecoado midiaticamente. Ao mesmo tempo, a maior conectividade digital juvenil na periferia e na universidade permitiu que ficassem sem aulas presenciais, e sustentou nossa colaboração para respondermos à crise. Por telefone ou online, os estudantes de Ensino Médio e nós, miniequipes de pesquisadoras(es) alocadas em cada território escolar, mantivemos o diálogo, frequentemente interagindo com a coordenação pedagógica, direção e professoras(es) envolvidas(os) com o projeto, discutindo e consolidando análises do que co-observávamos em cada território.

Nos primeiros 18 meses o diálogo e a escuta dependeram dessa interação desde as casas, onde todos estávamos isolados, mediados por telas e internet - de baixa qualidade para os jovens que moravam na periferia ^32^. A noção freiriana de decodificação das palavras até a práxis foi transposta para uma decodificação de cenas cotidianas de exposição aos diversos agravos que atravessavam o cotidiano dos adolescentes e jovens, na busca dos sentidos e significados locais que permitissem coproduzir práticas de prevenção ^2^ ^,^ ^5^ ^,^ ^13^ ^,^ ^15^ ^,^ ^33^. A releitura de *Pedagogia da Esperança: Um Reencontro com a Pedagogia do Oprimido* ^34^ foi especialmente inspiradora. Freire discute como situações de desastres e emergências podem paralisar a ação e insiste que tais situações (como secas e enchentes matadoras que o povo brasileiro sempre enfrentou) também mobilizam a inovação coletiva e solidária necessária para enfrentá-las, mobilizando a construção do que chama de “*inéditos viáveis*”; inovar exige sairmos do conforto das práticas conhecidas para “*coletivamente reinventar*”.

Percorríamos um “*terreno que se movia e mudava enquanto tentávamos atravessá-lo*”, como diria Burawoy ^35^ (p. 19) e a etnografia que descrevia cotidianos e cenários jamais vividos permaneceu online até outubro de 2021, quando a vacinação chegou à comunidade escolar.

Nesse período desenhamos abordagens que historicamente concebemos como psicossociais, psicoeducativas e dramatúrgicas, em contraste com a sociopsicológica-modeladora-de comportamentos, superando a educação para a prevenção que Freire chamaria de “*bancária*” ^5^ ^,^ ^36^. Com base na decodificação da vida cotidiana realizada nas sessões online com os jovens, compunha-se uma descrição de cenas de exposição a diferentes agravos (COVID-19, IST, gravidez não planejada, violências e sofrimento psicossocial) vividas ou observadas por estudantes agentes-jovens. Em seguida as ampliávamos para compreender como essas cenas expressavam o contexto compartilhado que estrutura as práticas de cada trajetória pessoal e desejo. Apoiando os adolescentes e jovens participantes, experimentávamos refazer a cena por meio de imaginação dirigida ^37^, buscando inéditos viáveis, para que as próximas cenas de interação fossem mais protegidas. A unidade para decodificação e intervenção eram os jovens concebidos como sujeitos de direitos em suas cenas da vida cotidiana ^5^ ^,^ ^15^ ^,^ ^33^.

Os primeiros meses de nossa vídeo-etnografia foi de investimento em um vínculo orgânico com os jovens, professores e dirigentes escolares para acompanhamento das iniciativas e dificuldades dos jovens para compreender e promover a prevenção em cada território, assim como pela busca de evidências sólidas sobre modos de transmissão e prevenção à COVID-19. Nesse tempo, agentes-jovens observavam o contexto doméstico mudar radicalmente e, pela TV e plataformas que acessavam, o mundo parado e assustado. Descreviam e discutiam experiências específicas na sua comunidade escolar e na vizinhança - isoladas nos territórios onde moravam ^26^ ^,^ ^38^. As meninas mais frequentemente “*observavam da janela*” e por meio dos relatos dos que saíam de casa. Grande parte dos meninos saiu para a rua ocupada por homens - masculinidade estimulada pelo presidente Bolsonaro. Liberados do trabalho doméstico e cuidado das crianças sem escola - que sobrecarregaram as meninas - cansavam dos jogos eletrônicos, procuravam um jogo de bola, ou eram os escolhidos pela família para arrumar trabalho, substituindo adultos desempregados. Nas conversas em que as meninas os cobravam por romper com o isolamento e expor os familiares mais suscetíveis, considerar as pessoas como “*conhecidas*” justificava ignorar o risco de se infectar ou transmitir o SARS-CoV-2 ao encontrar os amigos - “*diminuía o risco*” se era “*pessoa conhecida*”, como na aids.

No período sem aulas presenciais e sem vacinas, sustentamos reuniões na forma de rodas de conversa “*síncronas*” usando aplicativos de *meeting*, ou “*assíncronas*” usando o WhatsApp, além de grupos focais online sobre temas que frequentemente surgiam ao mesmo tempo em vários territórios (Figura 1). A interação online com os agentes-jovens permitiu que a decodificação de cenas significativas, experimentadas ou observadas por eles, promovessem a reflexão sobre temas expressivos dos sofrimentos associados à vida cotidiana, que seguia acirrando a desigualdade de gênero nas esferas de trabalho doméstico e emprego, e o “assédio”, o “racismo”, a “violência policial”. Era complexa e dinâmica a elaboração que justificava romper a “quarentena”, como nomeavam, frequentemente “riam de nervoso”.

**Figura 1:** No WhatsApp, garoto expressa seus sentimentos sobre a saída da “quarentena” para trabalhar.

Nota: ilustração retirada de grupo de WhatsApp, sem autoria identificada, sem título e sem data.

Concomitantemente ao movimento *Me Too* nos Estados Unidos, observamos um aumento das denúncias de assédio perpetrado nas escolas por professores, funcionários e colegas ^39^: o distanciamento físico não impediu que continuasse acontecendo. Debatemos o caso do professor que se masturbou durante uma aula online e o de homens (desconhecidos) que invadiram salas virtuais fazendo gestos obscenos e mostrando o pênis na câmera.

Nesse contexto dominado pela extrema direita, a violência policial aumentava fortemente nas periferias, a ponto de o Supremo Tribunal Federal (STF) acatar pedido de limitação das ações policiais nas favelas durante a pandemia ^40^. Paralelamente, nas rodas de conversa online essa violência policial suscitou debates sobre opressão racial que derivaram em conversas sobre a discriminação que os jovens presenciavam diretamente. Discutiam o cenário escolar onde professores negros são minoria e os funcionários negros estão na limpeza e no apoio técnico. O “racismo estrutural” discutido por *influencers* passou a interpretar a falta de oportunidades de formação e de emprego.

As experiências de racismo, assédio e violências baseadas em gênero, somadas ao luto - 30% dos respondentes ao questionário em 2022 perderam vizinhos e parentes para a COVID-19 - demandaram ações de acolhimento ao expressivo aumento de sofrimento mental que já identificávamos em 2019 ^26^. Compreendemos melhor as necessidades de acolhimento do que nomeamos e concebemos como sofrimento psicossocial quando encontramos nas discriminações e violências oriundas do racismo, machismo e LGBTfobia as suas fontes mais significativas ^41^ ^,^ ^42^.

Mediados por celulares e telas, supervisionamos em encontros semanais os agentes-jovens de prevenção. Avaliamos, passo a passo, o processo de produção e implementação das “*oficinas online de prevenção de COVID-19*” acompanhando as ações que adolescentes e jovens produziram: materiais muito criativos sobre a importância de se ficar em casa, usar máscaras, vacinar-se. Por 18 meses produziram mensagens, *memes*, pesquisas de pequeno porte, vídeos, *lives* temáticas e pôsteres disseminados por WhatsApp, Instagram, Facebook e YouTube (Figura 2). Sustentamos, assim, o diálogo de agentes-jovens com os seus pares e o que definiram como “público”: colegas de escola, amigos, parentes e vizinhos.

**Figura 2:** *Memes*, vídeos e pôsteres divulgando atividades produzidas pelos jovens.

Nota: ilustrações retiradas de grupo de WhatsApp, sem autoria identificada, sem título e sem data.

O tema orientação sexual e identidade de gênero “ficava quente” quando discutimos resultados dos questionários - aplicado em 2019, debatido online em 2020, e do aplicado em 2022 - que confirmavam a proporção significativa de jovens que se declararam não heterossexuais, pessoas trans, não bináries e em dúvida. O conservadorismo das famílias foi indicado como fonte de sofrimento de jovens LGBTQIA+ em casa e um dos motivos fortes no processo dinâmico de “romper a quarentena” (sair de casa e do isolamento físico recomendado).

Sobre a discriminação na escola (diziam “bullying”), ressaltaram que o “tom de brincadeira” dificultava a denúncia e a resistência à violação de direitos baseados no racismo e no processo de assumir identidades sexuais e de gênero não tradicionais, uma experiência incrustrada na trajetória escolar. Essa forma de discriminação, ressaltaram, causa sofrimento a ser enfrentado como “um problema sério”.

Conversar online sobre sexualidade e práticas preventivas foi difícil, mesmo quando os agentes-jovens aprenderam a proteger a intimidade desligando vídeo, câmeras e/ou som no ambiente domiciliar. Quando finalmente retomamos o tema da sexualidade na volta ao ensino presencial no final de 2021, os estudantes gostaram muito: “*Ufa, chega de COVID!*”.

Mas só estranheza aparecia no lugar do constrangimento quando iniciávamos a conversa sobre o “sexo na pandemia” e sobre quais seriam as práticas interessantes e aceitáveis de prevenção. Sexo protegido parecia “de outro mundo”: usar máscara na transa sexual? Esse tema provocou gargalhadas desde quando perguntávamos sobre as práticas preventivas utilizadas na primeira e última relação sexual no questionário de 2022. As cenas descritas incluíram “teste para COVID-19 antes do sexo”, “banho e higiene corporal antes do sexo” - até então uma prática comum para “depois do sexo” e pouco eficaz para a prevenção de IST. Sexo seguro só em casa com “ficantes”/namorados, tornados “fixos” como nos primeiros meses da COVID-19 - sexo em casa na ausência dos pais ou com o seu consentimento. Usar camisinha, pílula? “*Tinha isso de graça no posto de saúde... ainda tem? Tá lotado de COVID!*”.

Presencialmente realizamos grupos focais sobre o impacto da COVID-19 na vida deles, quando definiram a vida exclusivamente online em frases como: “*no tempo do apocalipse zumbi*”. Faziam fortes referências ao cansaço e à dificuldade de encarnar, de modo permanente, a prevenção à COVID-19 ainda em curso, sinalizando as dificuldades da prevenção concomitante, na mesma cena, de agravos resultantes de outras pandemias em curso - sífilis e HIV - e do sofrimento psicossocial já interpretado pela comunidade escolar como “pandêmico”.

“*Quando eu peguei depressão*” foi a expressão que traduzia a sensação entre adolescentes e jovens em tempos de sindemia que reforçava a sua compreensão do caráter epidêmico de eventos de sofrimento psíquico/mental, mal-estar psicológico traduzido como se fosse um evento viral e se espalhasse como tal. Seguimos registrando as sensações de excesso de algo que os estudantes identificam como “ansiedade” e medos exacerbados que se acumulavam ^38^ ^,^ ^42^, interpelando professores próximos dos alunos, que não sentiam ter “capacitação para o tema” e passavam por experiências de sofrimento semelhantes.

Todo o trabalho de campo realizado com e por agentes-jovens - rodas de conversa sobre cenas vividas ou observadas, oficinas, grupos focais e observação participante da vida na vizinhança ou nos diferentes cenários das escolas na volta às aulas, além dos dados colhidos dos questionários e o debate sobre seus resultados - indicaram que os amigos e colegas eram a fonte mais importante e eficiente de apoio ao que a imprensa já nomeava de “epidemia de transtornos mentais” e que compreendemos a partir de uma concepção psicossocial dialogando com as perspectivas de saúde mental baseadas em direitos humanos ^43^.

Como já discutimos em outros artigos ^41^ ^,^ ^42^, os questionários indicaram taxas de mal-estar psicológico (que entendemos como sofrimento psicossocial) acima da média mundial. O cuidado da saúde mental foi a opção privilegiada por estudantes quando perguntávamos sobre as necessidades e demandas prioritárias às unidades de saúde no território - embora reclamassem das unidades especializadas em saúde mental, porque a fila de espera era demorada e o atendimento, quase sempre em grupo, não era o que esperavam.

## Da prevenção combinada à prevenção integral

Atravessados pela emergência da COVID-19, comprometidos e provocados pela memória da resposta à aids, promovemos o distanciamento social, a testagem para COVID-19 e o uso correto de máscaras como prevenção para a circulação e encontros inevitáveis, ou desejados. Em 2021, estimulamos a vacinação. Em 2022, quando realizávamos oficinas presenciais de prevenção de gravidez e ISTs (sífilis e aids), a mpox assombrou especialmente os jovens LGBTQIA+ e investigamos com mais detalhe o aumento do sofrimento psicossocial que já observávamos em 2019 ^26^. Em 2023-2024 as escolas voltaram a lidar com a dengue.

Nesse contexto sindêmico, buscamos a inovação no terreno instável que percorríamos e a importância da perspectiva da integralidade se evidenciava para a prevenção. Em meio ao modo polissêmico e polimórfico com que esse princípio doutrinário do SUS vem sendo incorporado na sua construção ^44^, assumimos, baseados na Teoria do Processo de Trabalho em Saúde ^45^ e de seu correlato conceito de Cuidado em Saúde ^46^, uma concepção de integralidade baseada em quatro eixos de (re)construção das práticas de saúde: (i) apreensão das necessidades de saúde de forma ampliada para além das demandas estritamente biomédicas e como uma totalidade, frente à qual devem fazer sentido as respostas oferecidas pelas ações de saúde; (ii) integração, nas finalidades das ações, da promoção da saúde, prevenção de agravos, tratamento e reabilitação; (iii) estabelecimento de articulações interdisciplinares e intersetoriais para o melhor alcance das finalidades do cuidado; e (iv) favorecimento de contextos de interação dialógica, plural e construtiva entre os diversos sujeitos envolvidos nas ações de saúde ^47^.

Apesar de bastante discutido no plano da organização das ações de cuidado no SUS, no campo da prevenção pouco se tem refletido sobre a operacionalização do princípio da integralidade. Ações de prevenção têm sido desenvolvidas segundo uma lógica verticalizada, centrada em um agravo à saúde específico, sem considerar contextos sociais e programáticos que determinam a vulnerabilização das pessoas e grupos sociais e abstraindo outros agravos e demandas de saúde coexistentes nas mesmas cenas da vida cotidiana.

A adoção da denominação “prevenção integral” expressa o esforço para avançar no manejo das sinergias entre agravos, para compreender cada uma das necessidades e demandas amplamente ressignificadas e o lidar cotidiano de forma igualmente reconstruída com cada uma. Ou seja, as três dimensões analíticas da vulnerabilidade (pessoal, social e programática) e o horizonte normativo dos direitos humanos devem nos mover não de forma a justapor ou hierarquizar necessidades e demandas, mas, sim, acionar capacidades de resposta na medida e na direção indicada pelos adolescentes e jovens, pelo seu significado prático e pelo modo como se interpelam mutuamente no seu agir cotidiano.

Nesse sentido, buscamos abordar dinâmica e integradamente eventos que se sobrepunham sinergicamente - COVID-19, IST/aids, gravidez indesejada, violências, e sofrimento psicossocial. Em 2022, quando conseguimos nos reunir presencialmente, implementamos essa tecnologia de educação preventiva no que viemos a chamar de “oficina de prevenção integral”. Planejada para seis encontros, a oficina visava decodificar cenas vivas de interação em que discutíamos quando e como usar máscaras, preservativos, anticoncepcionais e contracepção de emergência, PEP (profilaxia pós-exposição) e PrEP. Os agentes-jovens produziram com massa de farinha e sal o corpo sexual e reprodutivo, colocaram e tiraram camisinhas masculinas de um pepino/cenoura ou as camisinhas internas nos modelos 3D, além de localizar num mapa de cada território onde aconteciam as cenas de maior exposição e como acessar insumos de prevenção, autotestes para aids e COVID-19, e vacinação contra COVID e o HPV. Nas seis sessões imaginaram o seu modo de encarnar a prevenção e buscar acolhimento para mal-estar psicológico.

Ao tomarmos a cena como unidade interpretativa da vulnerabilização aos agravos que sindemicamente ameaçam a saúde física e mental dos adolescentes e jovens, que consideramos implicadas densamente nos contextos e cenários de seu viver cotidiano, esse recurso foi novamente produtivo para problematizar a vulnerabilização social e, nas intervenções, para apreender a mútua interpelação dos agravos e das respostas práticas viáveis e desejáveis para eles.

A recomposição das cenas de vulnerabilização, lidas sob a ótica de um cuidado em saúde orientado pelo horizonte de proteção e promoção dos direitos, dá visibilidade a sujeitos, cenários e tramas que contextualizam objetivos possíveis e providos de sentido prático para as ações de promoção da saúde e prevenção. Assim, atentos aos contextos de intersubjetividade atravessados por determinantes estruturais (condições materiais, relações de poder, culturas), assim como aos afetos e às narrativas presentes neles e que nos interconectam, tornamo-nos mais capazes de dialogar com os saberes tecnocientíficos e práticos na direção de uma prevenção efetivamente orientada pela integralidade, conforme buscamos representar na Figura 3.

**Figura 3:** Cuidado, integralidade, vulnerabilidade e direitos humanos.

Avançamos desde a proposta de prevenção combinada às IST e à aids que caminhávamos para implementar, porque mais uma vez fomos desafiados pelos aspectos interconectados e subjacentes a uma oferta de recursos preventivos apenas aparentemente equitativa e equivalente em suas implicações práticas.

A proposta de prevenção combinada surgiu no limiar do contexto de retrocessos políticos que assistimos na resposta à aids no Brasil e internacionalmente, quando falar sobre sexualidade, ou dialogar com as pessoas sobre sua experiência sexual, nos termos delas, saiu do foco da prevenção, crescentemente biomedicalizada. A “prevenção combinada” ^48^ ^,^ ^49^ começa com uma tentativa bem intencionada de vincular métodos de prevenção biomédica à tradição da prevenção chamada de “comportamental” - que preferimos chamar de “psicoeducativas” ou de “abordagem psicossocial” ^5^ ^,^ ^36^. Considera as diferenças de poder e culturais, ressalta a determinação socioestrutural da saúde e da doença ^31^. Sua implementação, entretanto, foi abrindo mão, como fez o governo brasileiro da época, de disputar com o discurso conservador e religioso que interdita abordagens na escola da sexualidade baseada em direitos humanos, especialmente com crianças e adolescentes. Muitos profissionais na educação e na saúde, e mesmo adolescentes e jovens, acharam vantajoso poder evitar esse tipo de investimento e substituir a conversa por “pílulas receitadas” para a prevenção.

Sem desconsiderar a relevância de dispormos de um leque variado de recursos preventivos a serem disponibilizados para a prevenção no SUS, não podemos desconhecer os contextos - históricos, econômicos, políticos e culturais - nos quais tecnologias de saúde são produzidas ^50^, inclusive as de prevenção. Nesse sentido, é preciso estarmos atentos não apenas às diferentes possibilidades e interesses no acesso aos diversos métodos e insumos de prevenção em cada contexto particular, mas também buscar clareza sobre as implicações ideológicas e políticas de cada uma dessas opções. É especialmente importante ressaltar o risco da adesão acrítica, e muitas vezes inadvertida, à lógica individualista de uma “universalização fracionária”, isto é, à concepção, ideologicamente enviesada, de que se trata de oferecer a todos os recursos disponíveis para a prevenção, cuja utilização e os resultados serão, porém, atribuição e responsabilidade exclusiva de cada um ^23^.

Ao longo do projeto, ao construirmos a tecnologia da prevenção integral, passamos a visar a coconstrução de competências transversais para a prevenção e a promoção da saúde integral - deles, de colegas e amigos, nas interações psicossociais no território -, buscando:

(a) Superar a noção de inevitabilidade dos agravos à saúde e mobilizar os estudantes como sujeitos de direitos - à escolarização que alimentava seus planos de estudo e trabalho futuros; à saúde integral; à alimentação e moradia de qualidade; à não discriminação com base na cor, gênero, orientação sexual, características físicas etc.;

(b) Ter acesso ao melhor consenso técnico-científico, aprender a atualizar informações - sobre como se prevenir, como ter acesso aos insumos - para “encarná-las” em cenas cotidianas de maior ou menor exposição às pandemias em curso;

(c) Compreender que as experiências mudam de acordo com cada cena cotidiana e seu cenário, atentos para decodificar cada cena de interação psicossocial.

Ao lidar com a pandemia de agravos à saúde mental, mantivemos a perspectiva baseada em direitos humanos ressaltada por organismos internacionais ^43^, e começamos a enfrentar o desafio de pensar a prevenção do sofrimento psicossocial também como tarefa coletiva - da escola e de projetos comunitários dedicados a adolescentes e jovens - criticando sua medicalização e individualização excessiva.

Nos encontros quase semanais com estudantes, depois da vacinação e volta às aulas, a experiência com a COVID-19 era sintetizada em frases como essa: “*a pandemia acabou, mas a discriminação e a violência nunca param*”. Essas formulações produziram uma leitura da realidade por quem a vive e que, em diálogo com a equipe de pesquisa que apoiava a decodificação de suas cenas e cenários e promovia a prevenção, construiu um conhecimento comprometido com o horizonte da igualdade e da justiça.

Encontros com professores(as) e familiares para discutir resultados completaram interpretações e informações sobre cenas vivas, contribuindo para sustentar a factibilidade do projeto. Relevante foi observar que muitos pais e educadores, quando eram adolescentes e alunos do Ensino Médio, participaram de programas de prevenção da aids e educação sexual na escola baseados em direitos humanos como o “Prevenção Também se Ensina” ^51^. Confirmamos como, mesmo em contextos de retrocesso e negligência, memórias de respostas a uma crise sanitária, como a da aids, integram histórias comunitárias e profissionais, indicando caminhos para a promoção da saúde em um território.

Ou seja, garantimos experiências bem-sucedidas de responder à sinergia entre agravos e promover a prevenção integral para mitigar a vulnerabilidades valorizando a participação (princípio organizativo do SUS) como articuladora dos princípios de ações em saúde baseados em direitos humanos a não discriminação, a acessibilidade, disponibilidade e aceitabilidade de serviços de saúde e das ações da sociedade civil quando as pessoas receberam informações em linguagem adequada baseadas em evidências técnico-cientificas para tomar decisões bem-informadas, respeitando sua privacidade e a confidencialidade ^15^ ^,^ ^26^.

Nesse contexto sindêmico inaugurado pela COVID-19 em 2020, reafirmamos a produtividade do quadro dos direitos humanos para antecipar e mitigar a vulnerabilidade em territórios socialmente periferizados - a COVID-19 não se restringiria à zona oeste de São Paulo onde turistas de classe média que estiveram na Itália e países europeus chegavam trazendo o vírus. Confirmamos a máxima de que quanto maior a desigualdade, maior a violação de direitos e maior a vulnerabilidade à infecção, ao adoecimento e ao sofrimento psicossocial, legenda das sessões de discussão e capacitação para a prevenção, de profissionais e dos segmentos e territórios que abordamos neste quadro há duas décadas.

## Considerações finais

Este projeto foi um dos que apoiaram uma resposta social brasileira à COVID-19 baseada em direitos humanos e que resistiu como pôde ao desfecho evidentemente trágico - ainda não totalmente calculado em função do desmonte das tarefas históricas do Ministério da Saúde entre 2018 e 2022 - da violação de direitos fundamentais, das políticas negacionistas e negligentes articuladas pelo Governo Federal nos quatro anos ocupados por políticos que desprezam abertamente os direitos humanos. Mesmo com a eleição de governos cada vez mais conservadores, a articulação academia-sociedade civil na resposta à sindemia acirrada pela COVID-19 cobrou responsabilidades de gestores e validou ações programáticas sensíveis a inequidades estruturadas nas relações de gênero, classe e raça.

Fundamental nesse modelo de prevenção integral é investir na compreensão da resistência à desigualdade e iniquidade locais, sofisticada pela participação da comunidade no território e na sua capacitação. Os agentes-jovens de prevenção que acompanhamos lidaram com a polarização e a agressividade da extrema direita e setores conservadores movidos pelo pânico moral que obstaculizavam respostas com uma perspectiva interseccional à sindemia baseadas em direitos humanos. Assim, aprendemos que conversas públicas sobre como prevenir violência, abuso e assédio sexual, facilitam a conversa com conservadores que não aderem completamente à extrema direita que lidera o “pânico moral”, tal como parte das igrejas neopentecostais ativas nos territórios. Traduzidos para a linguagem da violação dos direitos humanos, esses temas priorizados por conservadores apoiaram a compreensão da relação indissociável entre maior ou menor vulnerabilidade e desrespeito ou proteção de direitos humanos, aumentando a possibilidade de colaboração comunitária nesse quadro.

A vida online não apenas exigiu a criatividade coletiva em metodologias inéditas e viáveis, como estimulou a criatividade dessa geração de adolescentes e jovens, que nasceu em tempos de internet, para coproduzir materiais de prevenção disseminados preferencialmente pelo Instagram e WhatsApp. Ressaltou, mais uma vez, a necessidade de os saberes práticos - como a experiência cotidiana de adolescentes e jovens em lidar consigo mesmos e com suas relações e contextos - serem ativamente buscados (e o método das cenas favorece isso), para que os necessários saberes tecnocientíficos sobre prevenção possam compor com eles o horizonte das ações de prevenção e não os substituir ou ignorá-los. Sem essa fusão de horizontes ^52^, nossos saberes e propostas de prevenção não fazem sentido, e as situações de prática que desejamos alcançar (relações, narrativas, afetos) permanecerão opacas para nós.

Assim aprendemos como - especialmente em tempos mediados pelo celular, aplicativos e redes sociais - não há empenho conservador que interdite o debate sobre sexualidades e gêneros (identidades e práticas) e elimine a experiência juvenil mobilizada pelo direito à não discriminação de corpos, identidades e desejos desvalorizados e estigmatizados ^53^.

Diferente da nossa experiência na resposta à aids, fortemente apoiada em movimentos identitários, a articulação academia-sociedade civil se organizou mais fortemente a partir de comunidades definidas por seus territórios, aprendizagem que não podemos desperdiçar ^54^. Embora limitada pelo contexto político, a colaboração e a criatividade da comunidade sustentou o reconhecimento dos adolescentes e jovens como sujeitos de direitos - à saúde, à prevenção na escola e à não discriminação - sem que suas diferenças (religiosas, étnico-raciais, de orientação sexual, de gênero) produzissem discriminação e exclusão.

Quando convidados a compor uma cena de exposição à COVID-19, os jovens inseriram o “consentimento” à interação e proximidade corporal, aprenderam a reconhecer insumos (máscaras, vacinação, testes) e suas formas de acessá-los (e os obstáculos para isso). Aumentaram a chance de também fazê-lo em relação às cenas de exposição a infecções sexualmente transmissíveis e a eventos não desejados como a gravidez e a violência entre parceiros. Ao conseguir decodificar cenas e cenários em que se vulnerabilizam, crescem em autonomia e produzem solidariedade social ao mesmo tempo em que identificam como, onde e quando acessar e usar (ou não) os diversos insumos e estratégias para prevenção integral.

Em linha com os aprendizados acima, nossa tese é que a tecnologia de prevenção integral delineada ao longo deste projeto e que seguimos avaliando propicia a cada pessoa reconhecer a si e aos demais como sujeitos de direitos e, nesse sentido, sempre imediatamente em relação com os grupos e contextos em que vive. Favorecer a integralidade na prevenção colabora para a compreensão da concomitância dos aspectos vulnerabilizadores nos contextos concretos em que vivemos e convivemos e da necessária articulação dos recursos para produzir respostas.

Mas, como diziam os adolescentes e jovens, que incluíram seu sofrimento psicossocial como prioritária necessidade na sindemia de pandemias em curso, complexo e difícil será pensar e agir “*enfrentando e prevenindo tudo ao mesmo tempo*”.

Nos territórios em que trabalhamos observamos que a cada cena cotidiana, e a cada novo desastre, pessoas, grupos e comunidades nos territórios acabam por escolher que “fios puxarão” da trama da prevenção e cuidado integral. Em outras palavras, a cada emergência que se avoluma, a cada peça desencaixada pelo desastre ambiental, a prioridade emergirá puxando o fio do mais saliente para cada pessoa e/ou território. Pode ser a fome, a falta de casa levada pelo fogo ou pela água, às vezes o sexo com prazer e protegido de doenças e sofrimento psicossocial ou o acesso à vacinação. No letramento para a prevenção, insistimos, ao puxar o fio não devemos perder o novelo da indivisibilidade dos direitos que garante mais dignidade à humanidade que compartilhamos - direito à alimentação, a acessar moradias e trabalhos decentes, a serviços de saúde de qualidade, da prevenção à reabilitação.

Finalmente, com a COVID-19 e a dengue de 2023/2024 mudamos de patamar na compreensão da gravidade dos efeitos da crise ambiental em curso, em especial sobre a saúde. Se nos espanta que “*o mundo não mudou!*” como os jovens e nós gostaríamos, não podemos voltar ao que fazíamos antes sem avançar e reformular. Para mudar o mundo, mesmo caminhando nesse terreno incerto, profissionais da prevenção precisam participar ativamente, com sua reflexão crítica, no debate sobre preparação/prontidão (*preparedness*) para as próximas e novas pandemias. Por incrível que possa parecer, a participação comunitária em cada território e a produção teórica e metodológica sobre prevenção estão ainda ausentes desse debate.

O caráter sindêmico das emergências sanitárias previstas em tempos de crise climática e ambiental, e a prontidão necessária ao seu enfrentamento, ampliaram os desafios da sustentabilidade de programas de prevenção e promoção da saúde em escolas. Nessa direção, ressaltamos que escolas sempre são lugares de referência em seu território e podem mitigar a vulnerabilidade a sofrimentos desnecessários e preveníveis, especialmente de crianças e jovens. Ou seja, investimentos em pesquisa e tecnologias sociais que garantam o direito à prevenção e a participação desde o território escolar deveriam fazer parte da preparação para crises sanitárias. Precisamos produzir o letramento para “decodificar” cenas cotidianas de interação social e sexual nos territórios onde moramos e circulamos, que permitirão antecipar a exposição e estimular a imaginação sobre o modo de “encarnar” a prevenção - da gravidez não planejada, do assédio e abuso sexual e, concomitantemente, a de doenças infecciosas como IST/aids, COVID-19, mpox, tubercole, dengue e as w, x, y, z que virão com secas, enchentes, desabamentos, interações entre diferentes biomas e a circulação rápida de pessoas pelos continentes.

Mesmo em contextos adversos, seguimos afiançando a produtividade dos princípios dos direitos humanos e de sua indivisibilidade, assim como da integralidade para ações de cuidado e de prevenção que reduzem vulnerabilidades, aspectos fundamentais para respondermos a esses tempos de “apocalipse zumbi”.
